# Morphological variability in *Lophyra flexuosa* (Fabricius, 1787) (Coleoptera, Cicindelidae) in desert countries is affected by sexual dimorphism and geographic aspect

**DOI:** 10.1002/ece3.8387

**Published:** 2021-11-24

**Authors:** Radomir Jaskuła, Axel Schwerk, Mateusz Płóciennik

**Affiliations:** ^1^ Department of Invertebrate Zoology & Hydrobiology Faculty of Biology & Environmental Protection University of Lodz Lodz Poland; ^2^ Department of Landscape Art Institute of Environmental Engineering Warsaw University of Life Sciences‐SGGW Warsaw Poland

**Keywords:** geographic variation in morphology, Maghreb region, Morocco, tiger beetles, Tunisia

## Abstract

*Lophyra flexuosa*, a eurytopic tiger beetle characterized by long phenological activity, wide geographic and altitudinal distribution, and occurring in the highest number of habitats among all Cicindelidae known from North Africa, was chosen to study its geographic variation in morphology and sexual dimorphism. Here, we found significant sexual dimorphism exhibited in larger body size of females and longer mandibles in males, which can be explained by different roles of particular sexes in courtship. Moreover, we recorded significant differences in body sizes between western and eastern Maghreb populations which could suggest genetic isolation between these populations. As the species is related to habitats placed close to the water reservoirs, which in the desert countries are under significant human pressure (including climate change), we expect a reduction of habitat occupied by this taxon. Therefore, the geographic morphological variability that we observe today in the tiger beetle *Lophyra flexuosa* in the future could lead to speciation.

## Graphical abstract

Our study is focused on *Lophyra flexuosa*, a tiger beetle species known as one of the most opportunistic species occurring in the southern part of the Mediterranean Region, which clearly suggests its high plasticity (including body parameters) according to climatic and habitat factors. In result, we evidence that populations of *L. flexuosa* from the western and eastern regions of Maghreb differ significantly what can confirm high plasticity of the species and/or can suggest genetic isolation between studied populations. Moreover, we note significant sexual dimorphism in this species and explain the size of particular sexes by their roles during courtship, the study is also one of the first attempts to statistically examine morphological variation in this beetle family in general.

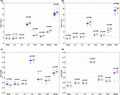

## 1 INTRODUCTION

Sexual dimorphism is a common phenomenon in insects, and it may be expressed in coloration (Thornhill & Alcock, 1983), defensive secretion (Attygale et al., 1991), or morphological features as dorsal punctures (Schwerk & Jaskuła, 2018), and/or body size and body shape (Thornhill & Alcock, 1983). Sexual selection is the main factor shaping differences in the body size and body shape between males and females in insects, even if such differences depend also on food availability during larval stages (Thornhill & Alcock, 1983). On the other hand, body size can differ geographically between particular populations of one animal species, both with latitude and altitude (e.g., Bergmann, 1847; Blanckenhorn et al., 2006; Partridge & Coyne, 1997; Stillwell et al., 2007) and/or as a result of separation by some geographic barriers (e.g., Stillwell & Fox, 2009; Wieczorek et al., 2017).

In the case of tiger beetles (Coleoptera: Cicindelidae), a family of predatory insects (Duran & Gough, 2020; López‐López & Vogler, 2017) with more than 2800 species distributed worldwide except polar regions and some oceanic islands (Cassola & Pearson, 2000; Wiesner, 2020), little is known about morphometric variability within particular species, except some data concerning sexual dimorphism. Generally, it is known that females are larger and wider than males (Espinoza‐Donoso et al., 2020; Jaskuła, 2005; Pearson & Vogler, 2001); in some genera different size and shape of labrum and mandibles between sexes was found too (Cassola & Bouyer, 2007; Jones & Conner, 2018; Kritsky & Simon, 1995). Doğan Sarikaya et al. (2020) noted differences in shape and size of head and pronotum between males and females of tiger beetles. Rarely, sexual differences can be observed also in the coloration of the body (Kippenhan, 1997; Pearson & Vogler, 2001) or only some parts of body are differently colored, for example, prothoracic tarsal pads (Palmer, 1981) or mandibles (Cassola & Bouyer, 2007; Pearson, 1988), which in the case of males are usually white, while in females they are darkly colored. Moreover, like all other Adephaga beetles, males of almost all tiger beetle species have ventral surfaces of the three to four tarsal segments of the first pair of legs, thickly covered with pads of setae, which is an adaptation to grasp and hold females during copulation (Stork, 1980)

The tiger beetle, *Lophyra flexuosa* (Fabricius, 1787), is widely distributed in western Palearctic, occurring from the Iberian Peninsula and Morocco in the west to Middle Asia in the east, with most of the localities known from the south Mediterranean region (Assmann et al., 2018; Jaskuła, 2015; Jaskuła & Rewicz, 2015; Jaskuła et al., 2015; Lisa, 2002; Matalin & Chikatunov, 2016; Putchkov & Matalin, 2003; Serrano, 2013; Wiesner, 2020). In Maghreb, the species is widespread and recognized as euryoecious, inhabiting the highest number of habitats among all known Cicindelidae species recorded from this area (Jaskuła, 2015; Jaskuła & Płóciennik, 2020). Moreover, it can be characterized both as species with the widest altitudinal distribution and the longest phenological activity among all tiger beetles known from this region (Jaskuła & Rewicz, 2015; Jaskuła et al., 2015).

Based on the wide distribution of *Lophyra flexuosa* and its large habitat spectrum, we hypothesized that the species should possibly show morphological variability observed not only in the sexual dimorphism in body size (which is regularly observed in tiger beetles) but also in the geographic variability (particular populations are often divided by geographic barriers, especially in mountain massifs and desert areas), which is commonly found in taxa characterized by large geographic ranges. Therefore, the aim of the following research was to test the following hypotheses:

1. Sexual dimorphism in *Lophyra flexuosa* is present in bigger body size of females and is observed in (most) morphological parameters. When standardizing the morphological parameters on total body length, some of the morphological parameters (e.g., mandibles because of their function in males during courtship) show higher values (i.e., are relatively bigger) in males than in females.
2. The average body size (values of the morphological parameters) of the individuals differs between the study sites located in different geographic regions and bioclimatic ecozones.

## 2 MATERIAL AND METHODS

### 2.1 Field sampling

Adult tiger beetles were collected by entomological hand nets during two TB‐Quest Expeditions organized to Morocco (March 2009) and Tunisia (March–April 2010). In total, 52 samples were collected including 20 in Morocco and 32 in Tunisia (Table 1). At each site the material was fixed in 96% ethanol for further morphological studies in the laboratory.

**TABLE 1:** Sampling localities for *Lophyra flexuosa* in Morocco and Tunisia

Sample code	Country	GPS co‐ordinate	Date	Sample code	Country	GPS co‐ordinate	Date
MO‐01	Morocco	N35.46514 W5.41840	06.04.2009	TN‐11	Tunisia	N32.98260 E9.63695	21.03.2010
MO‐02	Morocco	N35.24829 W5.33286	06.04.2009	TN‐13	Tunisia	N33.71373 E8.92086	22.03.2010
MO‐03	Morocco	N35.20274 W4.68136	22.04.2009	TN‐14	Tunisia	N33.91540 E8.13387	23.03.2010
MO‐05	Morocco	N35.11776 W4.17240	07.04.2009	TN‐15	Tunisia	N33.87572 E7.88200	23.03.2010
MO‐08	Morocco	N35.11579 W2.72510	08.04.2009	TN‐17	Tunisia	N34.37707 E7.91309	24.03.2010
MO‐10	Morocco	N35.10631 W2.36095	08.04.2009	TN‐18	Tunisia	N34.38284 E7.93288	24.03.2010
MO‐11	Morocco	N34.54452 W3.02568	09.04.2009	TN‐19	Tunisia	N34.39650 E8.83120	25.03.2010
MO‐13	Morocco	N34.24173 W3.31964	09.04.2009	TN‐20	Tunisia	N35.24704 E8.75249	26.03.2010
MO‐14	Morocco	N33.09332 W3.96598	10.04.2009	TN‐21	Tunisia	N35.20064 E8.87771	26.03.2010
MO‐16	Morocco	N32.87912 W4.23980	10.04.2009	TN‐22	Tunisia	N34.65176 E9.59818	26.03.2010
MO‐18	Morocco	N32.46998 W4.49573	11.04.2009	TN‐24	Tunisia	N34.4419 E10.27603	27.03.2010
MO‐19	Morocco	N31.67369 W4.19086	12.04.2009	TN‐29A	Tunisia	N35.67969 E10.1646	29.03.2010
MO‐21	Morocco	N31.13540 W6.34711	14.04.2009	TN‐30	Tunisia	N36.00233 E10.0399	30.03.2010
MO‐22	Morocco	N30.97698 W6.78775	14.04.2009	TN‐31	Tunisia	N36.02690 E9.42404	31.03.2010
MO‐23	Morocco	N30.91518 W6.90489	14.04.2009	TN‐32	Tunisia	N36.04641 E9.30721	31.03.2010
MO‐25	Morocco	N39.80286 W9.83609	14.04.2009	TN‐33B	Tunisia	N35.87081 E9.21404	31.03.2010
MO‐27	Morocco	N30.36317 W9.58335	20.04.2009	TN‐34A	Tunisia	N35.68057 E8.93391	31.03.2010
MO‐28	Morocco	N31.08910 W9.66635	21.04.2009	TN‐35	Tunisia	N35.80937 E8.56637	01.04.2010
MO‐29	Morocco	N31.98276 W9.32667	21.04.2009	TN‐36	Tunisia	N36.11506 E8.50126	01.04.2010
MO‐31	Morocco	N32.93012 W8.79314	21.04.2009	TN‐37	Tunisia	N36.11627 E8.64001	01.04.2010
TN‐02	Tunisia	N34.10833 E9.98197	18.03.2010	TN‐38	Tunisia	N36.21573 E8.62200	01.04.2010
TN‐03	Tunisia	N33.94027 E10.02673	18.03.2010	TN‐39	Tunisia	N36.41175 E8.55772	01.04.2010
TN‐05	Tunisia	N33.82404 E10.13745	18.03.2010	TN‐40	Tunisia	N36.40776 E8.75538	02.04.2010
TN‐06	Tunisia	N33.74928 E10.20916	18.03.2010	TN‐41	Tunisia	N36.64191 E8.70025	02.04.2010
TN‐07	Tunisia	N33.88635 E10.94381	19.03.2010	TN‐42A	Tunisia	N36.85951 E8.72154	02.04.2010
TN‐09	Tunisia	N33.72425 E10.95342	19.03.2010	TN‐44A	Tunisia	N36.64673 E9.60512	04.04.2010

### 2.2 Statistical analysis

**FIGURE 1:**
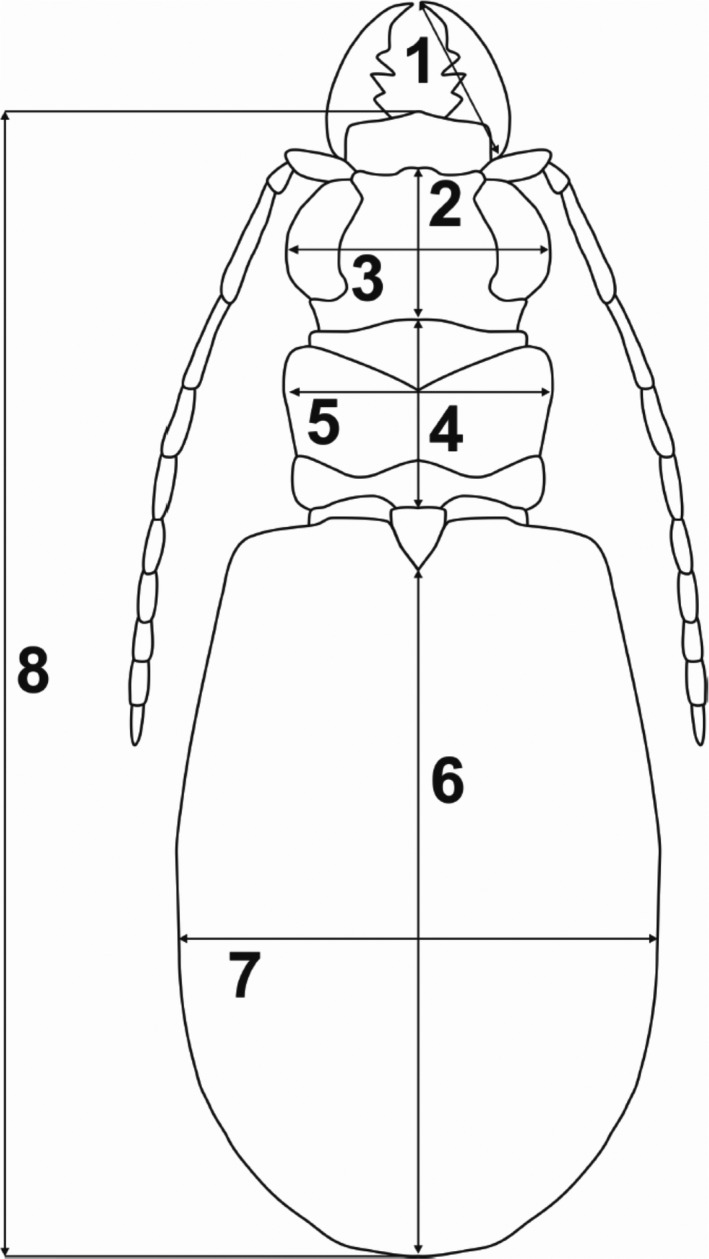
Body parameters measured in *Lophyra flexuosa*. 1—right mandible length (RML); 2—length of head (LH); 3—width of head (WH); 4—length of pronotum (LP); 5—pronotum width (WP); 6—length of elytra (LE); 7—maximum elytra width (MEW); 8—total body length (TBL)

To test the variation of morphometric traits, measurements of eight body parameters (Figure 1) were taken from all males and females used: right mandible length (RML); length of head (LH); width of head (WH); length of pronotum (LP); width of pronotum (WP); length of elytra (LE); maximum elytra width (MEW); and total body length (TBL). In total, 383 males (including 86 specimens from Morocco and 297 from Tunisia) and 352 females (including 75 individuals from Morocco and 222 from Tunisia) were measured.

In order to study sexual dimorphism, we first compared the measured values of the studied body parameters between males and females separately for each country. Next, right mandible length, length of head, width of head, pronotum length, maximum pronotum width, elytra length, and maximum elytra width were standardized on total body length by dividing the measured values by the total body length for each individual. These standardized values were also compared between males and females for both countries separately. In many cases Kolmogorov–Smirnov tests rejected normal distribution of the data. Therefore, the measured and standardized values were tested for statistically significant differences by applying non‐parametric Mann–Whitney *U* tests using TIBCO Statistica v. 13.3.

Non‐metric MultiDimensional Scaling (NMDS) was conducted to recognize variation in the morphology of North African *Lophyra flexuosa* populations. The analysis was conducted separately for males and females using Euclidean Distance similarity on normalized morphometric data. NMDS is a multi‐variate ordination technique that reflects a similarity between samples arranging them in multiple variables simplified to a two‐dimensional or three‐dimensional space. Additionally, SIMilarity PERcentage (SIMPER) analysis was conducted with normalized morphometric data and Euclidean Distance to recognize main body metrics that respond to the differentiation of Tunisian and Moroccan populations. Analysis Of SIMilarities (ANOSIM) with normalized morphometric data and Euclidean Distance was conducted to test the significance of differences between males and females from Moroccan and Tunisian populations separately. The NMDS, SIMPER, and ANOSIM were calculated with PRIMER 6 software (Clarke & Gorley, 2001).

To check if there is any relation between *L*. *flexuosa* body size and altitude, Spearman correlation was done.

## 3 RESULTS

**FIGURE 2:**
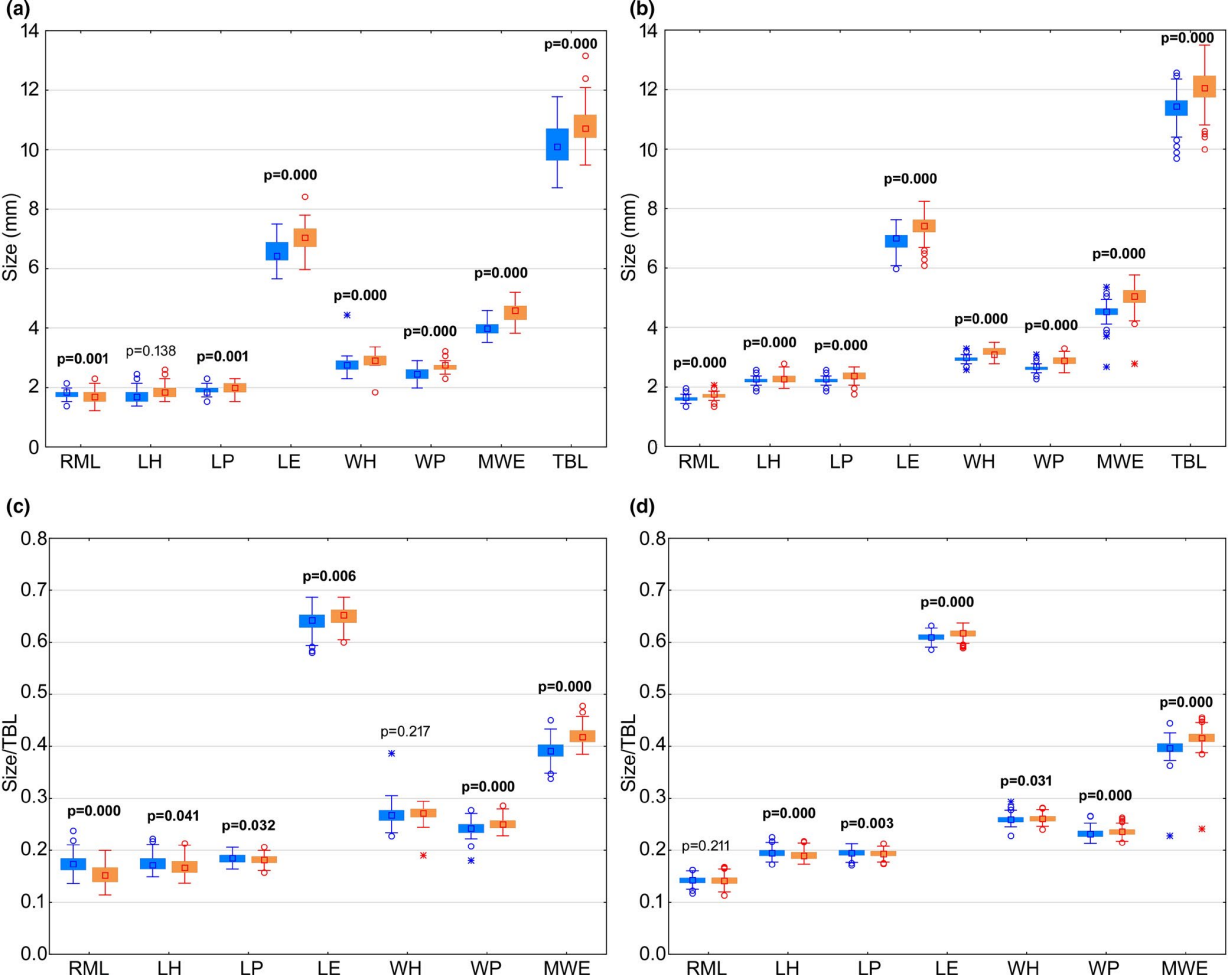
Box‐whisker plots for the measured values of selected body parameters for male and female individuals of *Lophyra flexuosa* in Morocco (a) and Tunisia (b) and for selected body parameters standardized on total body length (Size/TBL) for male and female individuals of *L*. *flexuosa* in Morocco (c) and Tunisia (d). The boxes represent the interquartile distances with median values drawn in. Whiskers indicate range of data with exception of outliers (distance from the edge of the box between 1.5 and 3 times of the box length, shown as circles) and extreme values (distance from the edge of the box more than three times the box length, shown as asterisks). Blue—males, red—females. Abbreviations of body parameters as in Figure 1 (Mann–Whitney *U* tests: significant *p*‐values are printed bold)

Comparison of the measured values of the body parameters of the individuals collected in Morocco (Figure 2a) revealed significantly higher median values of right mandible length (RML) in males. With the exception of length of head (LH), all other body parameters showed significantly higher median values in females. However, with respect to the measured values of individuals collected in Tunisia for all body parameters the median values were significantly higher in females (Figure 2b). When standardizing the body parameters on total body length the Moroccan individuals showed significantly higher median values in males for RML, LH, and length of pronotum (LP) (Figure 2c). With the exception of width of head (WH), all other body parameters had significantly higher median values in females. The standardized values for individuals from Tunisia revealed significantly higher median values in males for LH and LP (Figure 2d). RML had also higher median values in males, but the result was not significant. All other body parameters showed significantly higher median values in females.

**FIGURE 3:**
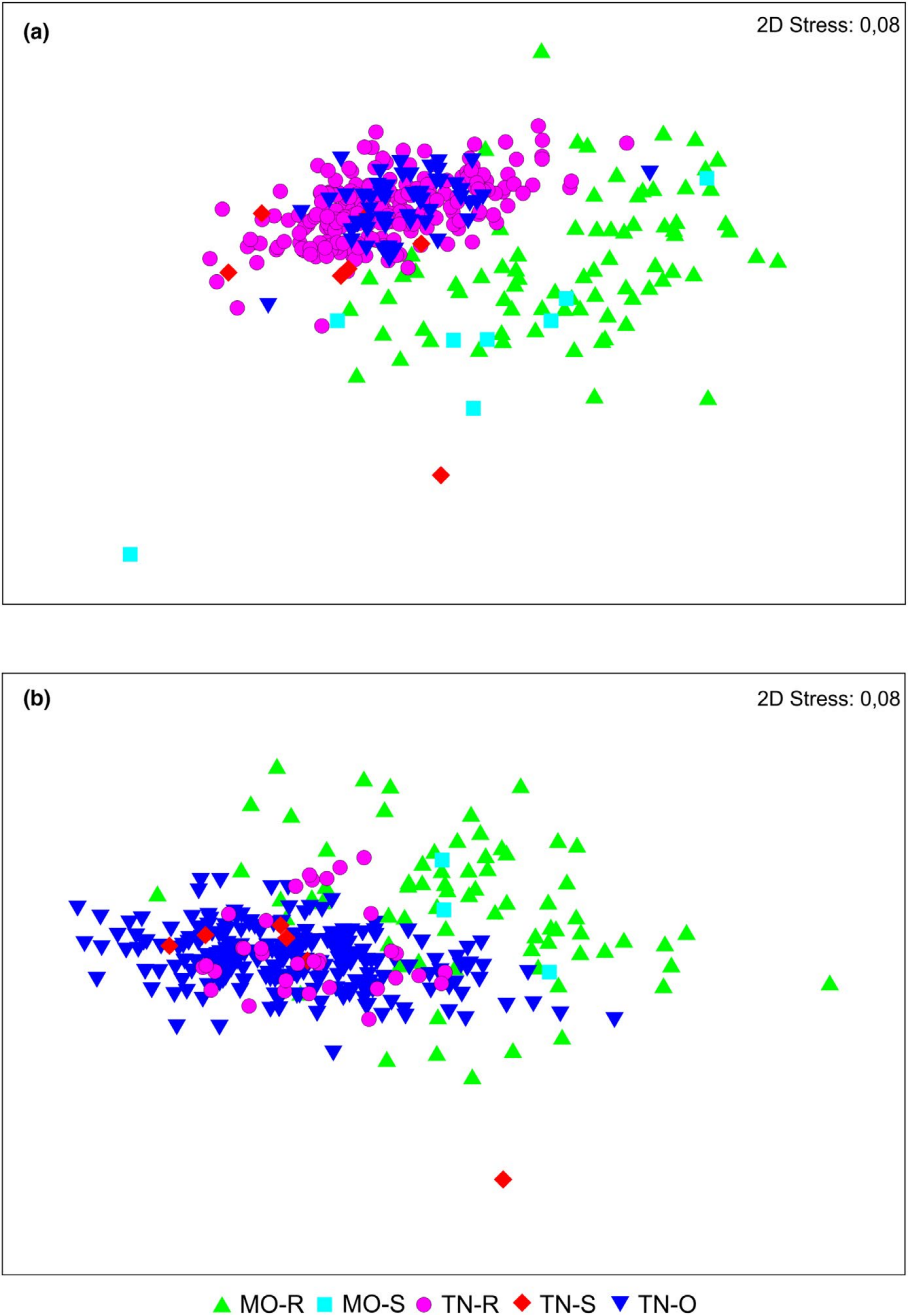
Results of NMDS analysis. a—males, b—females. Each symbol remarks singular specimen and its position depends on morphometric variables. The investigated male and female populations are divided according to countries and macrohabitats: TN, Tunisia; MO, Morocco; R, river banks; S, saltmarshes; O, oases

NMDS analysis (Figure 3) clearly separated the western and eastern Maghreb populations (from Morocco and from Tunisia respectively) for males and females. In both sexes, morphology is more unified in Tunisian populations (more in males then females) and more variable in Moroccan populations. *Lophyra flexuosa's* morphological variability did not reveal any clear pattern according to environmental factors like macrohabitats (Figure 3) and climate zonation (not illustrated). On the other hand, weak negative correlation between total body length (TBL) and altitude was observed for both sexes when the material from both countries was analyzed (Figure 5a,b; $r_{s\;\text{male}}=‐0.2470$, $r_{s\;\text{female}}=‐0.2110$) as well as only for the Moroccan population (in case of Tunisian beetles almost constant values were noted) (Figure 5c,d). The SIMPER analysis (Appendix S1) indicated that width of head (WH), right mandible length (RML), length of elytra (LE), and width of pronotum (WP) contributed the most for the Moroccan male population's distinctness, whereas width of pronotum (WP), length of elytra (LE), and maximum elytra length (MWE) contributed the most for the Tunisian male population's distinctness. The length of head (LH), total body length (TBL), and length of pronotum (LP) contributed the most to the Euclidean distance between the Moroccan and Tunisian male populations. In female populations, RML, WH, and LH contributed the most for the Moroccan population's distinctness, whereas WP, LE, and MWE contributed the most for the Tunisian population's distinctness. LH, LP, and TBL contributed the most to the Euclidean distance between the Moroccan and Tunisian male populations.

**FIGURE 4:**
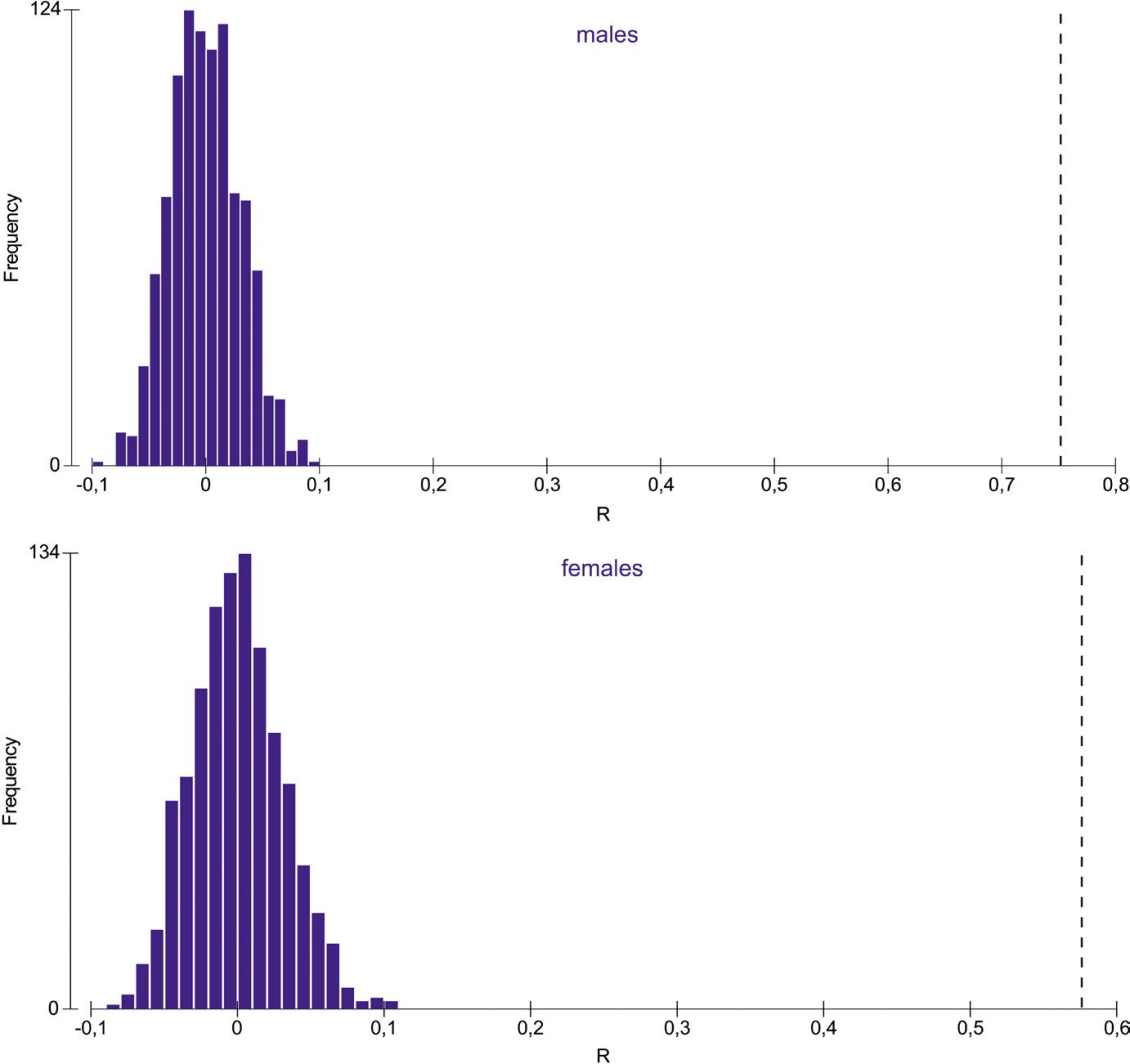
Results of ANOSIM analysis for the studied populations of *Lophyra flexuosa* from Tunisia and Morocco with distribution of R statistics

The ANOSIM analysis conducted with 999 permutations for males as well females indicated that male populations from Morocco and Tunisia were significantly different (*p* = .000) and clearly distinct (*R* = 0.753) (Figure 4). The female populations from Morocco and Tunisia were also significantly different (*p* = .000), but the difference was less distinct than for males (*R* = 0.577). The number of permuted statistics greater than or equal to Global R was 0 both for males and females.

**FIGURE 5:**
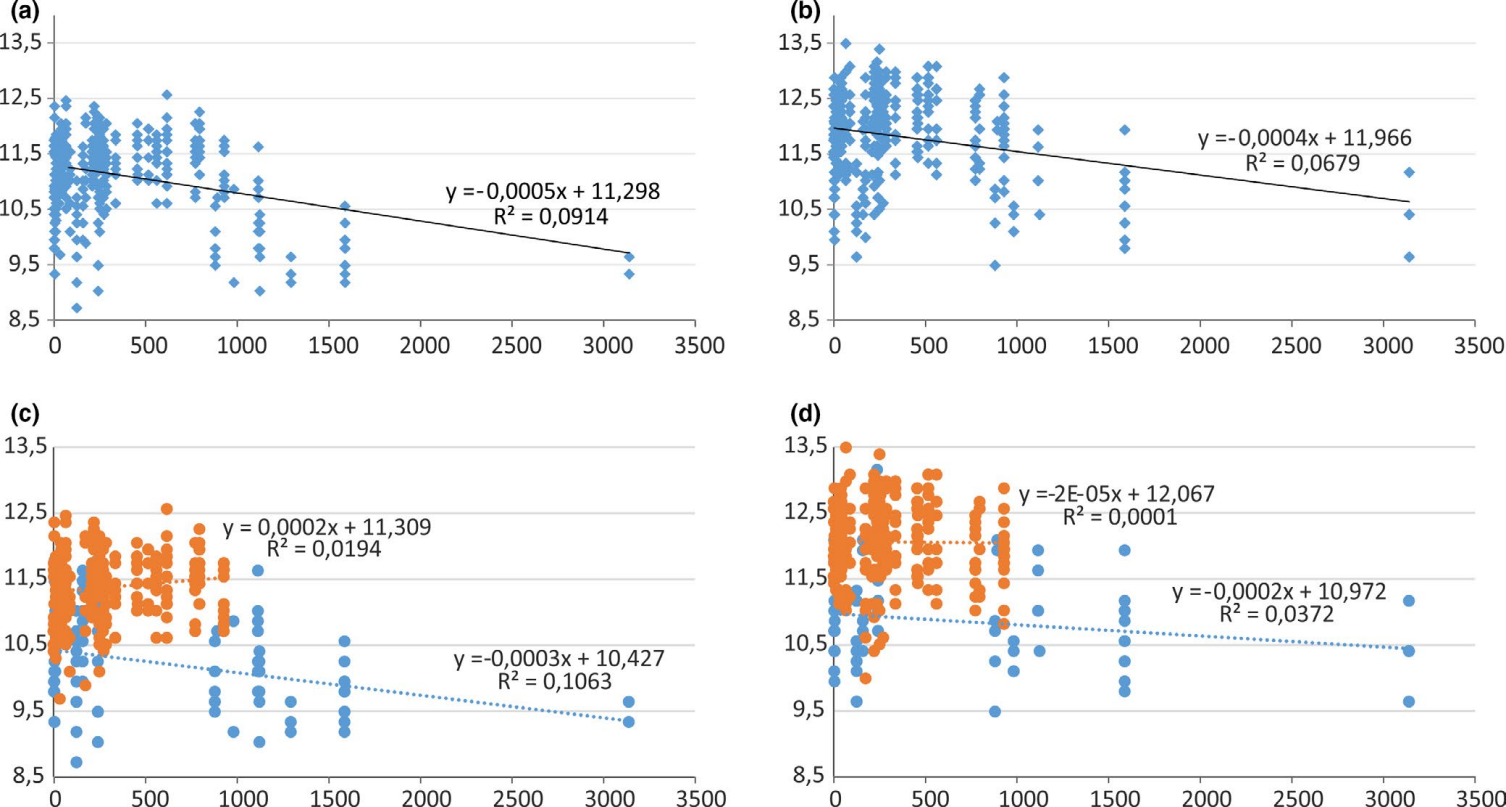
Plot of body size and altitude in the population of *Lophyra flexuosa* from Maghreb: all males versus altitude (a), all females versus altitude (b), separately males from Tunisia (orange dots) and Morocco (blue dots) versus altitude (c), separately females from Tunisia (orange dots) and Morocco (blue dots) versus altitude (d)

Both males and females were characterized by negative correlation between total body length and altitude when populations from Morocco as well as from the entire Maghreb region were analyzed. In contrast, the Tunisian population of *L*. *flexuosa* was characterized by constant (females) or almost constant values (males) (Figure 5).

## 4 DISCUSSION

As suggested by Losos and Miles (1994), morphology is determined by both genotype and phenotype, so it can provide insights into the phylogeny and ecology of a studied taxon and the selective pressures driving its evolution. Prior to our study, interspecific and intersexual body shape variations were observed only in a single tiger beetle species but not yet quantified for any *Lophyra* species nor for any North African Cicindelidae. Moreover, this study is the first attempt to statistically examine morphological variation in North African desert tiger beetles and one of the only few in this beetle family in general (e.g., Doğan Sarikaya et al., 2020; Espinoza‐Donoso et al., 2020; Franzen, 2007; Jaskuła, 2005; Pearson & Vogler, 2001).

In the studied populations of *Lophyra flexuosa* from the Maghreb region, females are bigger and wider than males (Figure 2), both in the case of the entire population and in each investigated country. This clearly suggests that such sexual dimorphism is characteristic for the entire species and does not depend on geographic region. Our findings confirm earlier studies on other Cicindelidae species (e.g., Jaskuła, 2005; Pearson & Vogler, 2001), with the exception of species classified in the Manticorini tribe in which males are characterized by bigger body size (Mareš, 2002). Although in tiger beetles differences in body size are also dependent on food availability during the larval stages (Pearson & Knisley, 1985), generally it is known as strongly connected with sex of the beetle and known as sexual dimorphism (Pearson & Vogler, 2001). The bigger body size found in females is usually explained by the role of this sex in mating behavior. As it was found in many different taxonomical insect groups, females invest much more energy in reproduction process than males, as a result a bigger size is much more beneficial for this sex (Thornhill & Alcock, 1983). First, females have to produce eggs, which need to be supplied in substances used in larval development, and second, they also need to find a good place to deposit them. These are among the most crucial conditions in females' post‐copulatory reproductive behavior as they determine developmental success of their embryos and as a consequence have significant impact on overall reproductive success. In contrast, males usually invest only their sperm, as a result their energetic costs during courtship are much smaller. All these elements of mating behavior can be easily found in tiger beetle species (Pearson & Vogler, 2001).

The bigger body size of females is often explained also by male choice during courtship. Although we know only a little about such behavior in Cicindelidae (even if some general casual observations are known for many species), including no detailed data about the mating behavior of the African population of *Lophyra flexuosa*, results of studies on many other insect groups clearly show that bigger females are preferred by males (e.g., Crespi, 1989; Gwynne, 1981; Harari et al., 1999; Johnson, 1992; Sota et al., 2000; Thornhill & Alcock, 1983). Such males' strategy can be easily explained if we note that bigger females can potentially produce and lay a higher number of eggs and/or they can supply the eggs in much more substances for better development of embryos. As a consequence, a male which will copulate with such kind of females may potentially increase his reproductive success (Thornhill & Alcock, 1983).

Although we noted that most of the measured body parameters had higher values in females, we also found that values of some parameters were higher in males of *Lophyra flexuosa*, especially when standardized on total body length (Figure 2), namely length of pronotum, length of head, and right mandible length. On the other hand, differences in shape, size, and even in colors of mouthparts in tiger beetles were noted earlier in some other Cicindelidae species as an example of significant sexual dimorphism. For example, Kritsky and Simon (1995) found smaller central teeth of mandibles and shorter labrum in some North American taxa. A shorter labrum was recorded also in case of, for example, the African genus *Neochila* by Cassola and Bouyer (2007), who noted also different coloration of mandibles and labrum between sexes, as well as in South American *Oxycheila* (Wiesner, 1999) and *Pseudochycheila* (Cassola, 1998). Much longer mandibles are characteristic also for males of all known members of the African tribe Manticorini (Mareš, 2002) and were found in a very common central European species *Cicindela hybrida* (Jaskuła, 2005). All these differences in size and shape of mandibles and labrum between males and females of tiger beetles can be explained by the role of these parts of mouthparts during courtship as smaller teeth of mandibles and shorter labrum allow males to better maintain and grasp the female's thorax during copulation (Pearson & Vogler, 2001). Moreover, as it was suggested in an earlier study (Jaskuła, 2005), longer mandibles and wider distance between their bases means greater length between the end parts of these organs, when mandibles are fully opened, what probably allows catching and grasping bigger females during mating, and as a consequence, help males with longer mandibles to increase their reproductive success. Mandible length has a special meaning, because, as in case of other Cicindelidae species (e.g., Gilbert, 1997; Rewicz & Jaskuła, 2018), also in *L*. *flexuosa*, mandibles are used by both sexes to catch and kill prey, as a consequence their size is sometimes mentioned as a very important factor determining types (and size) of prey during hunting behavior (Pearson & Mury, 1979). Different sizes and shapes of mandibles were also noted between tiger beetle species which co‐occur in one type of habitat as a possible way to reduce food competition between such species (e.g., Pearson & Juliano, 1991; Pearson & Mury, 1979; Satoh et al., 2003).

The analysis of body parameters allowed to recognize two morphological groups in *Lophyra flexuosa* which are separated geographically (Figures 3 and 4). Individuals occurring in eastern Maghreb (Tunisia) were noted as significantly larger in comparison with those from the western region (Morocco). Such a significant difference is rather unexpected as the population from entire North Africa (including both studied countries) is classified as one subspecies—*Lophyra flexuosa flexuosa* (Putchkov & Matalin, 2003; Wiesner, 2020). Although we had no opportunity to study specimens from Algeria, which is placed in central Maghreb just between Morocco and Tunisia and definitely they would be necessary to provide a full overview for the studied problem, we were able to note that the body size of *Lophyra flexuosa* shows a negative correlation with altitude, both in the case when the entire population was analyzed and the Moroccan population (Figure 5). Such results can be compared with some other studies as the body size of many animals, including insects, frequently varies also with altitude (e.g., Blanckenhorn et al., 2006; Partridge & Coyne, 1997; Stillwell et al., 2007). Moreover, as it was shown by Stillwell and Fox (2009) variation in body size, growth, and life‐history traits of ectotherms along altitudinal gradients is generally assumed to represent adaptation to local environmental conditions, especially to temperature. However, the degree to which the variation along such clines due to adaptation versus plasticity is still poorly studied and understood. On the other hand, we also found that altitude was not a factor which morphologically separated populations from Tunisia and Morocco. The significant differences observed in body size both in males and females suggest high plasticity of this species and/or a long‐term geographic isolation of both populations. This probably can be explained not only by the large geographic distance between them but also by the large number of natural barriers (especially particular mountain ranges of the Atlas Mts. as well as desert areas) (Blondel et al., 2010; Houérou, 2009). As noted above, an opportunity to study additional material from the area of Algeria probably would help to understand patterns of geographic variability in the population of *Lophyra flexuosa* in the entire Maghreb region. Especially future molecular analysis could help to understand if the observed morphological differences between Tunisian and Moroccan populations result from their genetic diversity like in the case of *Dromochorus* tiger beetle species noted by Duran et al. (2019) in North America. Individuals from the western population had relatively longer mandibles than individuals from Tunisia. Future research may also focus on studying what physical or biological factors, associated with body part size differences, change over the range. Trade‐offs between basic functions of mandible length (prey size selection and in case of males—grasping the female during copulation) might be impacted by such factors. On the other hand, our results are the first step to detect and quantify changes in the studied taxon even at the interspecific level. Although additional studies are needed (with molecular analysis and behavioral experiments if possible) earlier studies by, for example, Bookstein (1997), Alibert et al. (2001), Adams et al. (2004), Adams et al. (2013), or Espinoza‐Donoso et al. (2020) clearly suggest than modern morphometrics provide a fast, cheap, and accurate method for visualization of subtle shape changes between organisms and can be very useful for modern taxonomy.

## CONFLICT OF INTEREST

All authors declare no conflict of interest including any financial, personal, or other relationships with other people or organizations within 3 years from the beginning of the submitted work that could inappropriately influence, or be perceived to influence, their work. All authors declare that species studied in the text is not threatened or protected by law in any of the studied countries. Moreover, material was collected outside of any protected area as a result no special permissions were necessary during field work.

## AUTHOR CONTRIBUTIONS

**Radomir Jaskuła:** Conceptualization (lead); data curation (lead); formal analysis (lead); investigation (lead); methodology (lead); supervision (lead); writing–original draft (lead); writing–review and editing (lead). **Axel Schwerk:** Formal analysis (equal); writing–original draft (equal); writing–review and editing (equal). **Mateusz Płóciennik:** Formal analysis (equal); writing–original draft (equal); writing–review and editing (equal).

## Supporting information

Supplementary Material

## Data Availability

The file with raw data is available in the Dryad database under the following link: https://doi.org/10.5061/dryad.w0vt4b8pm.
