# Supplementary material for: Morphological variability in Lophyra flexuosa (Fabricius, 1787) (Coleoptera, Cicindelidae) in desert countries is affected by sexual dimorphism and geographic aspect
Source: Ecol Evol. 2021 Nov 24;11(23):17527–36. doi: 10.1002/ece3.8387 (PMC8668792; doi:10.1002/ece3.8387)
Supplement: Supplementary file 1 — Supplementary Material [file ECE3-11-17527-s001.docx]

SIMPER

Similarity Percentages - species contributions (MALES)

One-Way Analysis

*Data worksheet*

Name: Data4

Data type: Other

Sample selection: All

Variable selection: All

*Parameters*

Resemblance: D1 Euclidean distance

Cut off for low contributions: 90,00%

*Factor Groups*

Sample Tunisia-Morocoo

MO-19 M

MO-19 M

MO-19 M

MO-19 M

MO-19 M

MO-19 M

MO-18 M

MO-18 M

MO-18 M

MO-18 M

MO-18 M

MO-18 M

MO-18 M

MO-18 M

MO-18 M

MO-18 M

MO-29 M

MO-29 M

MO-14 M

MO-28 M

MO-28 M

MO-28 M

MO-28 M

MO-28 M

MO-28 M

MO-28 M

MO-28 M

MO-28 M

MO-11 M

MO-11 M

MO-11 M

MO-11 M

MO-08 M

MO-13 M

MO-13 M

MO-13 M

MO-08 M

MO-08 M

MO-08 M

MO-08 M

MO-08 M

MO-02 M

MO-02 M

MO-01 M

MO-01 M

MO-01 M

MO-01 M

MO-01 M

MO-01 M

MO-01 M

MO-05 M

MO-05 M

MA-10 M

MO-10 M

MO-10 M

MO-10 M

MO-03 M

MO-03 M

MO-03 M

MO-03 M

MO-01 M

MA-01 M

MO-01 M

MO-27 M

MO-27 M

MO-25 M

MO-25 M

MO-23 M

MO-23 M

MO-23 M

MO-23 M

MO-23 M

MO-23 M

MO-23 M

MO-22 M

MO-22 M

MO-22 M

MO-22 M

MO-22 M

MO-22 M

MO-22 M

MO-21 M

MO-21 M

MO-21 M

MO-16 M

MO-16 M

TN-18 T

TN-18 T

TN-02 T

TN-02 T

TN-02 T

TN-02 T

TN-02 T

TN-02 T

TN-02 T

TN-02 T

TN-02 T

TN-02 T

TN-02 T

TN-02 T

TN-02 T

TN-15 T

TN-15 T

TN-15 T

TN-15 T

TN-15 T

TN-15 T

TN-15 T

TN-15 T

TN-15 T

TN-15 T

TN-14 T

TN-14 T

TN-14 T

TN-14 T

TN-14 T

TN-14 T

TN-14 T

TN-14 T

TN-14 T

TN-14 T

TN-14 T

TN-14 T

TN-14 T

TN-14 T

TN-14 T

TN-22 T

TN-22 T

TN-22 T

TN-22 T

TN-22 T

TN-22 T

TN-22 T

TN-22 T

TN-22 T

TN-22 T

TN-22 T

TN-22 T

TN-22 T

TN-22 T

TN-22 T

TN-33B T

TN-33B T

TN-33B T

TN-33B T

TN-33B T

TN-33B T

TN-33B T

TN-33B T

TN-33B T

TN-33B T

TN-33B T

TN-33B T

TN-33B T

TN-33B T

TN-31 T

TN-31 T

TN-31 T

TN-31 T

TN-31 T

TN-13 T

TN-13 T

TN-13 T

TN-13 T

TN-13 T

TN-13 T

TN-13 T

TN-13 T

TN-13 T

TN-13 T

TN-13 T

TN-13 T

TN-13 T

TN-13 T

TN-13 T

TN-13 T

TN-05 T

TN-05 T

TN-06 T

TN-06 T

TN-06 T

TN-06 T

TN-06 T

TN-06 T

TN-06 T

TN-06 T

TN-06 T

TN-07 T

TN-07 T

TN-07 T

TN-07 T

TN-07 T

TN-32 T

TN-32 T

TN-32 T

TN-32 T

TN-32 T

TN-32 T

TN-32 T

TN-32 T

TN-31 T

TN-31 T

TN-31 T

TN-31 T

TN-31 T

TN-11 T

TN-11 T

TN-11 T

TN-11 T

TN-11 T

TN-11 T

TN-11 T

TN-11 T

TN-11 T

TN-11 T

TN-11 T

TN-11 T

TN-11 T

TN-11 T

TN-21 T

TN-21 T

TN-21 T

TN-21 T

TN-21 T

TN-21 T

TN-21 T

TN-21 T

TN-21 T

TN-21 T

TN-21 T

TN-21 T

TN-21 T

TN-21 T

TN-21 T

TN-21 T

TN-21 T

TN-21 T

TN-19 T

TN-19 T

TN-19 T

TN-19 T

TN-19 T

TN-19 T

TN-19 T

TN-19 T

TN-19 T

TN-03 T

TN-03 T

TN-03 T

TN-03 T

TN-03 T

TN-24 T

TN-24 T

TN-24 T

TN-24 T

TN-24 T

TN-24 T

TN-24 T

TN-34A T

TN-34A T

TN-34A T

TN-34A T

TN-34A T

TN-34A T

TN-34A T

TN-34A T

TN-34A T

TN-34A T

TN-34A T

TN-17 T

TN-17 T

TN-17 T

TN-17 T

TN-17 T

TN-17 T

TN-17 T

TN-17 T

TN-17 T

TN-17 T

TN-17 T

TN-20 T

TN-20 T

TN-20 T

TN-20 T

TN-20 T

TN-20 T

TN-20 T

TN-20 T

TN-20 T

TN-20 T

TN-20 T

TN-38 T

TN-38 T

TN-38 T

TN-38 T

TN-38 T

TN-38 T

TN-38 T

TN-38 T

TN-38 T

TN-38 T

TN-38 T

TN-38 T

TN-38 T

TN-38 T

TN-38 T

TN-37 T

TN-36 T

TN-36 T

TN-36 T

TN-36 T

TN-36 T

TN-36 T

TN-36 T

TN-36 T

TN-35 T

TN-35 T

TN-35 T

TN-35 T

TN-35 T

TN-35 T

TN-35 T

TN-35 T

TN-35 T

TN-35 T

TN-35 T

TN-35 T

TN-35 T

TN-35 T

TN-40 T

TN-40 T

TN-40 T

TN-40 T

TN-40 T

TN-40 T

TN-42A T

TN-39 T

TN-39 T

TN-44A T

TN-44A T

TN-44A T

TN-44A T

TN-44A T

TN-44A T

TN-44A T

TN-44A T

TN-44A T

TN-44A T

TN-44A T

TN-44A T

TN-44A T

TN-44A T

TN-44A T

TN-44A T

TN-44A T

TN-44A T

TN-41 T

TN-41 T

TN-41 T

TN-41 T

TN-41 T

TN-41 T

TN-41 T

TN-41 T

TN-41 T

TN-41 T

TN-41 T

TN-41 T

TN-41 T

TN-30 T

TN-30 T

TN-30 T

TN-30 T

TN-30 T

TN-30 T

TN-30 T

TN-29A T

TN-29A T

TN-29A T

TN-29A T

TN-29A T

TN-29A T

TN-29A T

*Group M*

Average squared distance = 9,93

Species Av.Value Av.Sq.Dist Sq.Dist/SD Contrib% Cum.%

MWE -1,21 0,673 0,47 6,77 6,77

LP -1,3 0,74 0,49 7,45 14,22

TBL -1,25 1,03 0,48 10,34 24,57

LH -1,38 1,11 0,46 11,19 35,76

WP -0,826 1,36 0,48 13,73 49,49

LE -0,845 1,37 0,49 13,75 63,24

RML 0,901 1,74 0,46 17,53 80,77

WH -0,852 1,91 0,20 19,23 100,00

*Group T*

Average squared distance = 3,88

Species Av.Value Av.Sq.Dist Sq.Dist/SD Contrib% Cum.%

LH 0,401 0,254 0,43 6,54 6,54

TBL 0,363 0,407 0,43 10,47 17,01

LP 0,377 0,444 0,45 11,44 28,45

WH 0,247 0,47 0,44 12,10 40,56

RML -0,261 0,486 0,46 12,52 53,08

MWE 0,351 0,547 0,26 14,10 67,18

LE 0,245 0,631 0,45 16,25 83,43

WP 0,239 0,643 0,43 16,57 100,00

*Groups M & T*

Average squared distance = 29,62

Group M Group T

Variable Av.Value Av.Value Av.Sq.Dist Sq.Dist/SD Contrib% Cum.%

LH -1,38 0,401 4,54 1,22 15,31 15,31

TBL -1,25 0,363 4,03 1,03 13,62 28,94

LP -1,3 0,377 3,99 1,04 13,46 42,40

MWE -1,21 0,351 3,65 1,05 12,33 54,73

WH -0,852 0,247 3,56 0,46 12,03 66,76

RML 0,901 -0,261 3,56 0,82 12,01 78,77

LE -0,845 0,245 3,17 0,83 10,69 89,46

WP -0,826 0,239 3,12 0,76 10,54 100,00

SIMPER

Similarity Percentages - species contributions (FEMALE)

One-Way Analysis

*Data worksheet*

Name: Data4

Data type: Other

Sample selection: All

Variable selection: All

*Parameters*

Resemblance: D1 Euclidean distance

Cut off for low contributions: 90,00%

*Factor Groups*

Sample Tunisia-Morocco

MO-18 M

MO-19 M

MO-19 M

MO-19 M

MO-19 M

MO-18 M

MO-18 M

MO-18 M

MO-18 M

MO-18 M

MO-18 M

MO-18 M

MO-18 M

MO-18 M

MO-18 M

MO-16 M

MO-16 M

MO-16 M

MO-16 M

MO-28 M

MO-28 M

MO-28 M

MO-31 M

MO-31 M

MO-31 M

MO-14 M

MO-14 M

MO-28 M

MO-28 M

MO-11 M

MO-11 M

MO-08 M

MO-08 M

MO-08 M

MO-13 M

MO-13 M

MO-13 M

MO-08 M

MO-08 M

MO-02 M

MO-02 M

MO-02 M

MO-02 M

MO-01 M

MO-01 M

MO-01 M

MO-01 M

MO-01 M

MO-01 M

MO-01 M

MO-05 M

MO-05 M

MO-05 M

MO-10 M

MO-10 M

MO-10 M

MO-10 M

MO-10 M

MO-10 M

MO-10 M

MO-03 M

MO-03 M

MO-03 M

MO-01 M

MO-01 M

MO-01 M

MO-01 M

MO-01 M

MO-01 M

MO-25 M

MO-22 M

MO-23 M

MO-23 M

MO-23 M

MO-23 M

TN-02 T

TN-02 T

TN-02 T

TN-02 T

TN-02 T

TN-02 T

TN-02 T

TN-02 T

TN-02 T

TN-02 T

TN-02 T

TN-02 T

TN-02 T

TN-15 T

TN-15 T

TN-15 T

TN-15 T

TN-15 T

TN-15 T

TN-15 T

TN-15 T

TN-15 T

TN-15 T

TN-15 T

TN-15 T

TN-15 T

TN-14 T

TN-14 T

TN-14 T

TN-14 T

TN-14 T

TN-22 T

TN-22 T

TN-22 T

TN-22 T

TN-33B T

TN-33B T

TN-33B T

TN-33B T

TN-33B T

TN-33B T

TN-31 T

TN-31 T

TN-31 T

TN-31 T

TN-31 T

TN-13 T

TN-13 T

TN-13 T

TN-13 T

TN-13 T

TN-13 T

TN-13 T

TN-13 T

TN-13 T

TN-13 T

TN-13 T

TN-05 T

TN-05 T

TN-06 T

TN-06 T

TN-06 T

TN-06 T

TN-06 T

TN-06 T

TN-07 T

TN-07 T

TN-07 T

TN-07 T

TN-32 T

TN-32 T

TN-32 T

TN-32 T

TN-32 T

TN-32 T

TN-32 T

TN-32 T

TN-32 T

TN-32 T

TN-32 T

TN-32 T

TN-32 T

TN-32 T

TN-32 T

TN-32 T

TN-32 T

TN-31 T

TN-31 T

TN-31 T

TN-31 T

TN-31 T

TN-11 T

TN-11 T

TN-11 T

TN-11 T

TN-11 T

TN-11 T

TN-21 T

TN-21 T

TN-21 T

TN-21 T

TN-21 T

TN-21 T

TN-21 T

TN-21 T

TN-21 T

TN-19 T

TN-19 T

TN-19 T

TN-19 T

TN-19 T

TN-19 T

TN-19 T

TN-19 T

TN-19 T

TN-19 T

TN-19 T

TN-19 T

TN-24 T

TN-24 T

TN-24 T

TN-24 T

TN-24 T

TN-09 T

TN-34A T

TN-34A T

TN-34A T

TN-34A T

TN-34A T

TN-34A T

TN-34A T

TN-34A T

TN-34A T

TN-34A T

TN-34A T

TN-34A T

TN-34A T

TN-34A T

TN-34A T

TN-17 T

TN-17 T

TN-17 T

TN-17 T

TN-17 T

TN-17 T

TN-17 T

TN-17 T

TN-20 T

TN-20 T

TN-20 T

TN-20 T

TN-20 T

TN-20 T

TN-20 T

TN-20 T

TN-20 T

TN-20 T

TN-20 T

TN-20 T

TN-20 T

TN-20 T

TN-20 T

TN-20 T

TN-20 T

TN-20 T

TN-20 T

TN-20 T

TN-36 T

TN-36 T

TN-36 T

TN-36 T

TN-36 T

TN-36 T

TN-36 T

TN-36 T

TN-36 T

TN-36 T

TN-36 T

TN-36 T

TN-36 T

TN-38 T

TN-38 T

TN-38 T

TN-38 T

TN-38 T

TN-38 T

TN-38 T

TN-38 T

TN-38 T

TN-37 T

TN-37 T

TN-37 T

TN-37 T

TN-37 T

TN-37 T

TN-37 T

TN-37 T

TN-35 T

TN-35 T

TN-35 T

TN-35 T

TN-35 T

TN-35 T

TN-35 T

TN-35 T

TN-35 T

TN-35 T

TN-35 T

TN-40 T

TN-40 T

TN-40 T

TN-40 T

TN-40 T

TN-40 T

TN-40 T

TN-40 T

TN-40 T

TN-40 T

TN-40 T

TN-40 T

TN-40 T

TN-40 T

TN-40 T

TN-41 T

TN-41 T

TN-41 T

TN-41 T

TN-41 T

TN-41 T

TN-41 T

TN-41 T

TN-41 T

TN-41 T

TN-41 T

TN-41 T

TN-41 T

TN-44A T

TN-44A T

TN-44A T

TN-44A T

TN-44A T

TN-44A T

TN-44A T

TN-44A T

TN-44A T

TN-44A T

TN-44A T

TN-39 T

TN-39 T

TN-39 T

TN-39 T

TN-39 T

TN-29A T

TN-29A T

TN-29A T

TN-29A T

TN-29A T

TN-29A T

TN-29A T

TN-29A T

TN-29A T

TN-29A T

TN-29A T

TN-29A T

TN-29A T

TN-29A T

TN-29A T

TN-30 T

TN-30 T

TN-30 T

TN-30 T

TN-30 T

TN-30 T

TN-30 T

TN-30 T

TN-30 T

TN-30 T

*Group M*

Average squared distance = 8,83

Species Av.Value Av.Sq.Dist Sq.Dist/SD Contrib% Cum.%

MWE -0,985 0,632 0,45 7,15 7,15

LP -1,36 0,647 0,46 7,33 14,48

TBL -1,22 0,867 0,44 9,82 24,30

LE -0,779 1,04 0,41 11,73 36,03

WP -0,596 1,04 0,43 11,80 47,83

LH -1,36 1,12 0,45 12,66 60,49

WH -0,935 1,21 0,25 13,74 74,23

RML -0,221 2,28 0,47 25,77 100,00

*Group T*

Average squared distance = 5,03

Species Av.Value Av.Sq.Dist Sq.Dist/SD Contrib% Cum.%

LH 0,368 0,334 0,43 6,64 6,64

LP 0,369 0,456 0,42 9,07 15,72

TBL 0,33 0,527 0,44 10,48 26,20

WH 0,253 0,644 0,45 12,82 39,02

RML 5,98E-2 0,645 0,46 12,82 51,84

MWE 0,267 0,767 0,29 15,26 67,10

LE 0,211 0,784 0,42 15,60 82,70

WP 0,161 0,87 0,46 17,30 100,00

*Groups M & T*

Average squared distance = 26,72

Group M Group T

Variable Av.Value Av.Value Av.Sq.Dist Sq.Dist/SD Contrib% Cum.%

LH -1,36 0,368 4,42 1,14 16,54 16,54

LP -1,36 0,369 4,1 1,05 15,33 31,87

TBL -1,22 0,33 3,78 1,02 14,14 46,00

WH -0,935 0,253 3,25 0,47 12,17 58,17

RML -0,221 5,98E-2 2,97 0,77 11,10 69,28

MWE -0,985 0,267 2,96 0,84 11,06 80,34

LE -0,779 0,211 2,78 0,78 10,42 90,76
